# Is interim ^18^F-fluoride PET/CT a predictor of outcomes after radium-223 therapy?

**DOI:** 10.1590/0100-3984.2017.0178

**Published:** 2019

**Authors:** Elba Etchebehere, Ana Emília Brito, Kalevi Kairemo, Eric Rohren, John Araujo, Homer Macapinlac

**Affiliations:** 1 Universidade Estadual de Campinas (Unicamp), Campinas, SP, Brazil.; 2 Real Hospital Português de Beneficência em Pernambuco - Real Nuclear, Recife, PE, Brazil.; 3 The University of Texas MD Anderson Cancer Center, Houston, TX, USA.

**Keywords:** Sodium fluoride, Positron-emission tomography/methods, Tomography, X-ray computed/methods, Prostatic neoplasms, Radium-223, Bone neoplasms/diagnostic imaging, Tumor burden, Fluoreto de sódio, Tomografia por emissão de pósitrons/métodos, Tomografia computadorizada/métodos, Neoplasias da próstata, Rádio-223, Neoplasias ósseas/diagnóstico por imagem, Carga tumoral

## Abstract

**Objective:**

To determine whether an interim ^18^F-fluoride positron-emission tomography/computed tomography (PET/CT) study performed after the third cycle of radium-223 dichloride (^223^RaCl_2_) therapy is able to identify patients that will not respond to treatment.

**Materials and Methods:**

We retrospectively reviewed 34 histologically confirmed cases of hormone-refractory prostate cancer with bone metastasis in patients submitted to ^223^RaCl_2_ therapy. All of the patients underwent baseline and interim ^18^F-fluoride PET/CT studies. The interim study was performed immediately prior to the fourth cycle of ^223^RaCl_2_. The skeletal tumor burden-expressed as the total lesion fluoride uptake above a maximum standardized uptake value of 10 (TLF_10_)-was calculated for the baseline and the interim studies. The percent change in TLF_10_ between the baseline and interim studies (%TFL_10_) was calculated as follows: %TFL_10_ = interim TLF_10_ - baseline TLF_10_ / baseline TLF_10_. End points were overall survival, progression-free survival, and skeletal-related events.

**Results:**

The mean age of the patients was 72.4 ± 10.2 years (range, 43.3-88.8 years). The %TLF_10_ was not able to predict overall survival (*p* = 0.6320; hazard ratio [HR] = 0.753; 95% confidence interval [CI]: 0.236-2.401), progression-free survival (*p* = 0.5908; HR = 1.248; 95% CI: 0.557-2.797) nor time to a bone event (*p* = 0.5114; HR = 1.588; 95% CI: 0.399-6.312).

**Conclusion:**

The skeletal tumor burden on an interim ^18^F-fluoride PET/CT, performed after three cycles of ^223^RaCl_2_, is not able to predict overall survival, progression-free survival, or time to bone event, and should not be performed to monitor response at this time.

## Abstract (pt)

**Objetivo:**

Avaliar se o PET/CT interim com fluoreto-^18^F após a terceira dose da terapia com dicloreto de rádio-223 (^223^Ra) é capaz de identificar pacientes que não responderão ao tratamento.

**Materiais e Métodos:**

Revisamos, retrospectivamente, 34 pacientes com diagnóstico histológico de câncer de próstata refratários a hormonioterapia e com metástases ósseas que foram submetidos a ^223^Ra. Todos os pacientes foram submetidos a PET/CT com fluoreto-^18^F antes de iniciar o tratamento (basal) e imediatamente antes da quarta dose de ^223^Ra (interim). A carga tumoral esquelética (TLF_10_) foi calculada em ambos os exames da PET/CT com fluoreto-^18^F de cada paciente e foi determinada a alteração percentual na TLF_10_ entre eles (%TFL_10_ = TLF_10_ interim - TLF_10_ basal / TLF_10_ basal). Foram avaliados a sobrevida global, a sobrevida livre de progressão e o tempo para um evento ósseo.

**Resultados:**

A idade média dos pacientes foi 72,4 ± 10,2 anos (variação: 43,3-88,8 anos). A %TLF_10_ não foi capaz de predizer a sobrevida global (*p* = 0,6320; HR = 0,753; intervalo de confiança [IC] 95%: 0,236-2,101), a sobrevida livre de progressão (*p* = 0,5908; HR = 1,248; IC 95%: 0,557-2,797) nem o tempo para um evento ósseo (*p* = 0,5114; HR = 1,588; IC 95%: 0,399-6,312).

**Conclusão:**

A carga tumoral esquelética da PET/CT com fluoreto-^18^F realizada após três doses de ^223^Ra não é capaz de predizer sobrevida global, sobrevida livre de progressão ou tempo até um evento ósseo, e não deve ser realizada para monitorar a resposta ao tratamento desses pacientes, nesse momento.

## INTRODUCTION

Baseline whole-body ^18^F-fluoride PET/CT is ideal for staging and restaging prostate cancer and has been shown to be an independent prognostic imaging biomarker of patients undergoing radium-223 dichloride (^223^RaCl_2_) therapy^(^1^)^. However, although treatment with ^223^RaCl_2_ improves survival in prostate cancer patients^(^2^-^4^)^, not all patients respond to this therapy. It would be beneficial to identify nonresponders early in the course of treatment, thereby reducing morbidity and unnecessary costs.

After successful treatment of osteoblastic bone metastases, an osteoblastic reaction (flare) can occur, which increases bone uptake even in responsive cases. That can be confused with the osteoblastic reaction and inflammation that occur in response to tumor-associated growth factors during progression. This phenomenon has been well described in conventional bone scintigraphy, and that method is therefore not recommended for use as the sole means of determining the response to treatment^(^5^,^^6^^)^.

Although interim studies performed with ^18^F-FDG PET/CT can change the management of patients with a variety of cancer types^(^7^-^10^)^, the exact role of ^18^F-fluoride PET/CT in evaluating the early response to therapy (interim study) is not well established. The importance of ^18^F-fluoride PET/CT has extended beyond the diagnosis of metastases to the evaluation of optimal strategies for use in patients submitted to treatment with new therapeutic agents. Chemotherapy, hormone therapy, immunotherapy, and radionuclide therapies such as those involving ^223^RaCl_2_^(^11^)^ are costly approaches. Therefore, the ability to predict response, thereby avoiding overtreatment and reducing costs, will improve patient management. The purpose of this study was to determine whether an interim ^18^F-fluoride PET/CT study is able to evaluate treatment responses in prostate cancer patients submitted to ^223^RaCl_2_ therapy.

## MATERIALS AND METHODS

The local institutional review board approved this retrospective analysis (reference no. PA14-0848). We retrospectively reviewed histologically confirmed cases of hormone-refractory prostate cancer with bone metastasis in patients receiving ^223^RaCl_2_ therapy and undergoing two ^18^F-fluoride PET/CT studies-a baseline study and an interim study (immediately prior to the fourth cycle of ^223^RaCl_2_)-at our facility. All patients completed at least four cycles of ^223^RaCl_2_ (Xofigo; Bayer Pharma AG, Berlin, Germany), receiving intravenous infusions of 50 kBq/kg (1.4 µCi/kg) of ^223^RaCl_2_ at monthly intervals.

### ^18^F-fluoride PET/CT acquisition

^18^F-fluoride PET/CT images were acquired immediately prior to initiation of the ^223^RaCl_2_ therapy (baseline study) and immediately before the fourth cycle (interim study). True whole-body PET images were obtained 50-60 min after intravenous injection of 158-370 MBq of ^18^F-sodium fluoride in dedicated PET/CT scanners (Discovery STe, RX, or VCT; 16 or 64 channel; GE Healthcare, Milwaukee, WI, USA, or Siemens mCT Flow; 64 channel; Siemens Healthcare, Knoxville, TN, USA), and whole-body noncontrast CT scans were used for attenuation correction.

### ^18^F-fluoride PET/CT interpretation and quantification

Two board-certified nuclear medicine physicians evaluated baseline and interim ^18^F-fluoride PET/CT images. Visual and quantitative analyses were performed.

#### Visual analysis

In the visual analysis, we compared the baseline and interim studies, classifying the responses as follows:

- Complete response - Osteoblastic bone metastases identified in the baseline study no longer being present in the interim study.
- Partial response - Interim study showing decreased uptake in pre-existing bone metastases.
- Stable disease - No difference between the interim and baseline scans in terms of the uptake in pre-existing bone metastases.
- Progressive disease - Interim study showing an increase in the uptake or volume of a pre-existing bone metastases or new osteoblastic metastases.

The patients were followed for confirmation of these response classifications. The follow-up reference standards used in order to determine if the response classification was correct (i.e., to identify true-positive, true-negative, false-positive, and false-negative responses) included clinical parameters-such as clinical worsening, disease progression, bone events, and death; biochemical parameters-such as the levels of alkaline phosphatase (ALP) and prostate-specific antigen (PSA); and imaging findings-such as those obtained with ^18^F-fluoride PET/CT, ^18^F-FDG PET/CT, bone scans, or CT scans. On the interim ^18^F-fluoride PET/CT study, images that demonstrated stable disease, a partial response, or a complete response were all considered to represent a true-positive response to therapy if the reference standards also indicated that the patient had responded to therapy (no clinical worsening, progression, or increase in the levels of the biochemical markers) or a false-positive response to therapy if those same standards demonstrated progressive disease (new areas of disease, clinical worsening, or death). In contrast, images that demonstrated progressive disease were considered to represent a true-negative response to therapy if the reference standards also indicated that the patient had not responded to therapy and it was confirmed during follow-up that there was no response to therapy or a false-negative response to therapy (flare response) if those same standards demonstrated a response (no clinical worsening, progression, or increase in the levels of the biochemical markers).

#### Quantitative analysis

Using quantitative analysis, we determined the whole-body skeletal tumor burden in the baseline and interim ^18^F-fluoride PET/CT images. The skeletal tumor burden was determined after establishing the maximum standardized uptake value (SUV_max_) threshold ≥ 10 to exclude normal bone^(^12^)^. To that end, we initially obtained the volume (in milliliters) of total fluoride activity, defined as the fluoride tumor volume above an SUV_max_ of 10 (FTV_10_), within the volume of interest (VOI). The FTV_10_ calculation is equivalent to the metabolic tumor volume calculation used in ^18^F-FDG PET/CT studies. The total fluoride lesion uptake above an SUV_max_ of 10 (TLF_10_) was then calculated as the product of mean SUV_max_ × FTV_10_. The TLF_10_ is equivalent to the total lesion glycolysis used in ^18^F-FDG PET/CT studies. To evaluate the performance of interim ^18^F-fluoride PET/CT, the percent change in the skeletal tumor burden between the baseline and interim studies was calculated as follows:

%TLF_10_ = interim TLF_10_ - baseline TLF_10_ / baseline TFL_10_

### Statistical analyses

Categorical variables were expressed as absolute and relative frequencies, whereas continuous variables were expressed as mean ± standard deviation when presenting normal distribution and as median (minimum-maximum) when presenting non-normal distribution. All outcome measures were correlated with the %TFL_10_ values obtained. The primary end point was overall survival (OS), which was calculated from the first ^223^RaCl_2_ cycle to the date of death or last follow-up. Secondary end points were progression-free survival (PFS), time to a bone event (TTBE), and bone marrow failure (BMF). PFS was calculated from the first ^223^RaCl_2_ cycle to the date of progression, death, or last follow-up. The TTBE was calculated as the time from the date of the first ^223^RaCl_2_ cycle to the next bone event. Lastly, BMF was defined as the development of hematologic toxicity (World Health Organization grade 3 or 4), together with no recovery after six weeks or death due to BMF after the last ^223^RaCl_2_ cycle.

Kaplan-Meier survival curves were generated, and Cox proportional hazards regression was used in order to analyze predictors of survival. Backward stepwise selection was performed for multivariate Cox models. Logistic regression was used in order to model the odds of a bone event as a function of all of the PET variables. We used Spearman's correlation coefficient to assess the level of agreement between the PET variables. For the statistical analyses, we used the Statistical Analysis System, version 9.3 for Windows (SAS Institute Inc., Cary, NC, USA).

## RESULTS

We analyzed the cases of 34 patients, with a mean age of 72.4 ± 10.2 years (median, 72.5 years; range, 43.3-88.8 years) (Table 1), who had had prostate cancer for a mean of 6 ± 4 years (range, 2-20 years). The mean Gleason score was 7 ± 3. Prior to the initiation of ^223^RaCl_2_ therapy, 26.9% of the patients had received chemotherapy, 5% had received radiotherapy, 59% had received hormone therapy, and 9% had received blood transfusion. At the first ^223^RaCl_2_ cycle, the mean ALP was 193.9 IU/L and the mean PSA was 103.2 ng/mL. The median time of follow-up after the interim study was 28.1 months (range, 11-52 months). The 34 patients were submitted to a collective total of 179 ^223^RaCl_2_ cycles: 55.9% of the patients received six cycles of ^223^RaCl_2_; 14.7% received five cycles; and 29.4% received four cycles. The principal causes of treatment interruption were progression (in 44.4%), hematologic toxicity (in 17.8%), a significant decline of the Eastern Cooperative Oncology Group performance status (in 13.3%), and a bone event (in 2.2%).

**Table 1:** Demographic and clinical characteristics of 34 patients prior to 2^23^RaCl_2_ therapy.

Characteristic	Median	Range
Age	72.4	43.3-88.8
Prostate specific antigen (ng/mL)	103.2	2.1-761.1
Alkaline phosphatase (IU/L)	193.9	48.0-913.0
Hemoglobin (g/dL)	11.2	6.6-13.6
Platelets (K/µL)	215.8	114-413
Absolute neutrophils (K/µL)	5.7	1.5-21.4

### Visual analysis of interim ^18^F-fluoride PET/CT

**Figure 1:**
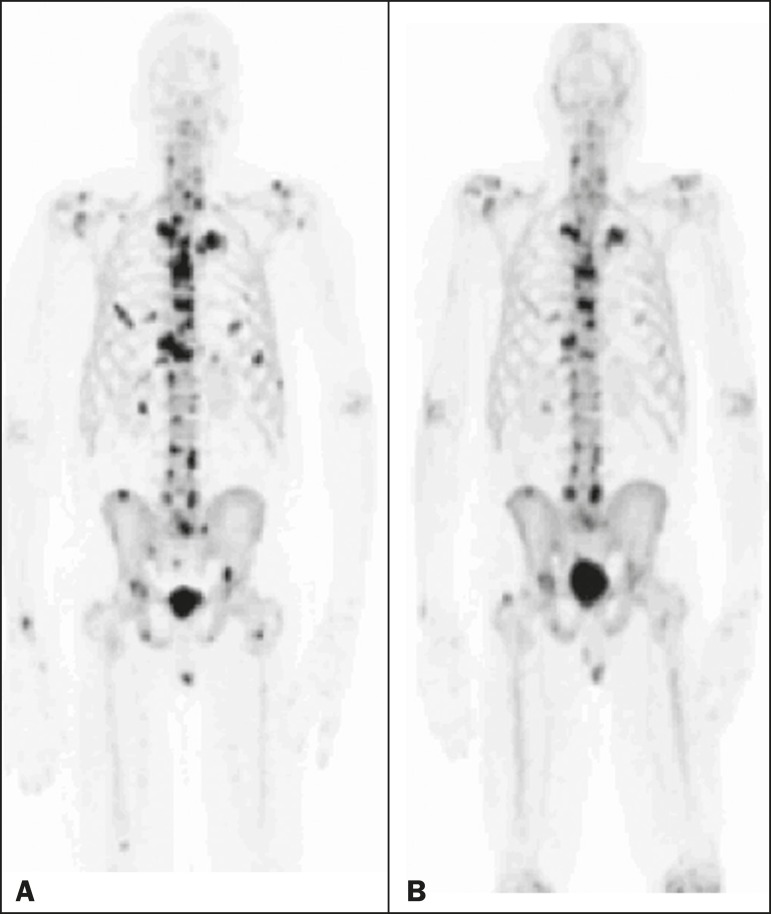
A patient with hormone-refractory prostate cancer, accompanied by bone metastasis, who showed a partial response to ^223^RaCl_2_, and the interim ^18^F-fluoride PET/CT study demonstrating a true-positive response. **A:** The baseline ^18^F-fluoride PET/CT study revealing widespread osteoblastic metastases. **B:** The interim ^18^F-fluoride PET/CT study, performed after the third ^223^RaCl_2_ cycle, showing a reduction in osteoblastic metastases, especially in the rib cage, pelvis, and right femur, consistent with a partial response to ^223^RaCl_2_. There was a 70% reduction in the %TLF_10_. During follow-up, the ALP levels dropped and no new bone lesions appeared. After the last ^223^RaCl_2_ cycle, the patient resumed enzalutamide to control lymph node metastases that had been present prior to the first ^223^RaCl_2_ cycle.

**Figure 2:**
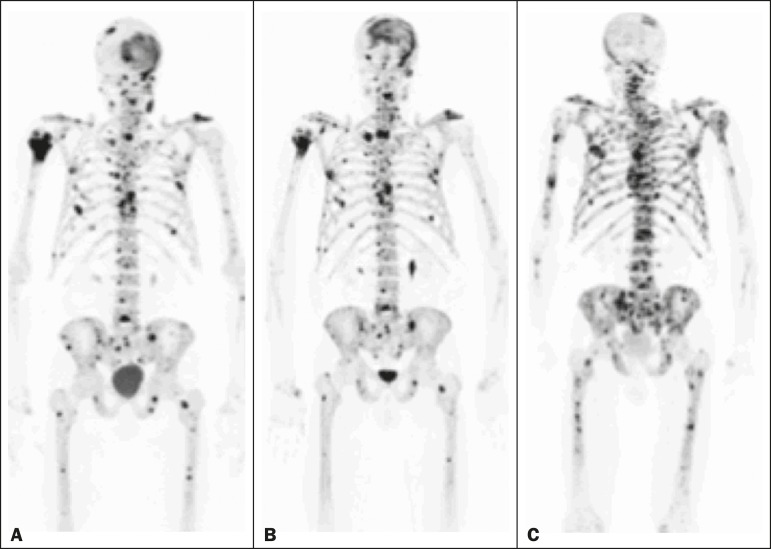
A patient with hormonerefractory prostate cancer, accompanied by bone metastasis, who showed progression during ^223^RaCl_2_ therapy but was categorized as a false-positive case on the basis of the imaging findings. **A:** The baseline ^18^F-fluoride PET/ CT study showing osteoblastic metastases. **B:** The interim ^18^Ffluoride PET/CT study, performed after the third ^223^RaCl_2_ cycle, showing a slight reduction in uptake by the known osteoblastic metastases and no new lesions, consistent with a partial response. Although the %TLF_10_ decreased by 44%, the PSA and ALP levels continued to rise and there was rapid progression of the bone metastases. Therefore, the patient was started on cyclophosphamide and subsequently on dasatinib. C: A follow-up ^18^F-fluoride PET/ CT study, conducted after the sixth ^223^RaCl_2_ cycle, showed wide- A B C spread osteoblastic metastases.

**Figure 3:**
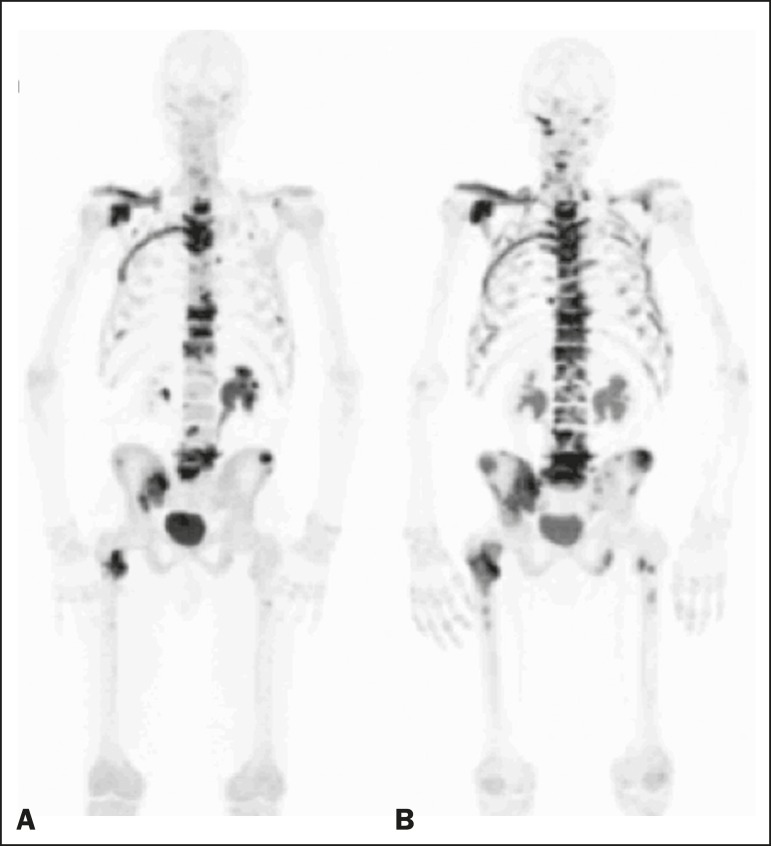
A patient with hormone-refractory prostate cancer, accompanied by bone metastasis, who showed progression during ^223^RaCl_2_ therapy. **A:** The baseline ^18^F-fluoride PET/CT study showing widespread osteoblastic metastases. **B:** The interim ^18^F-fluoride PET/CT study, performed after the third ^223^RaCl_2_ c ycle, s howing i ncreased u ptake i n t he k nown o steoblastic metastases and new lesions, especially in the pelvis, consistent with progression. The %TLF_10_ increased by 104%; PSA and ALP levels continued to rise; new bone metastases developed; a liver metastasis developed; and there was further enlargement of previously enlarged lymph nodes. The patient started a new chemotherapy regimen but died eight months after the last ^223^RaCl_2_ cycle.

**Figure 4:**
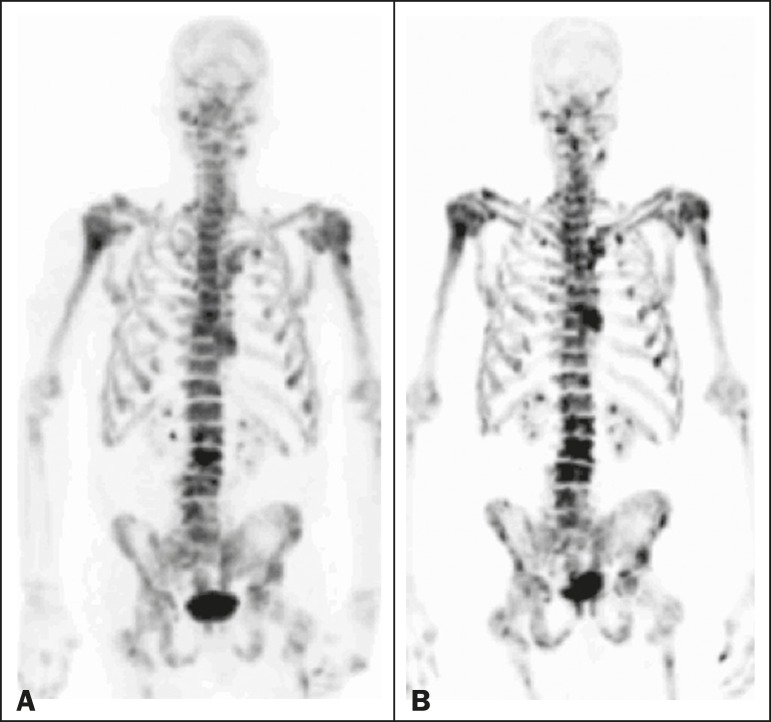
A patient with hormone-refractory prostate cancer, accompanied by bone metastasis, who responded to ^223^RaCl_2_ but was categorized as a falsenegative case on the basis of the imaging findings. **A:** The baseline ^18^F-fluoride PET/CT study showing osteoblastic metastases. **B:** The interim ^18^F-fluoride PET/CT study, performed after the third ^223^RaCl_2_ cycle, showing increased uptake in the known osteoblastic metastases but no new lesions. Although that pattern is consistent with progression (with a %TLF_10_ increase of 65%), the PSA and ALP dropped remarkably, after which the patient responded and was stable at 12 months after the last ^223^RaCl_2_ cycle. Therefore, the images were clearly due to a flare (false-negative) response.

A complete response was not perceived in any of the interim ^18^F-fluoride PET/CT studies or on the basis of the follow-up reference standards. A partial response was identified in 16 (47%) of the patients in the interim ^18^F-fluoride PET/CT studies (Figure 1), and the reference standards demonstrated that a partial response had indeed been achieved in eight of those patients (true-positive cases), whereas the other eight patients had progressed (false-positive cases), as shown in Figure 2. Stable disease was noted in five (15%) of the patients in the interim ^18^F-fluoride PET/CT studies, although only three of those patients were categorized as true-positive cases (showing stable disease or a partial response), whereas the two remaining patients progressed. Progressive disease was identified in 13 (38%) of the patients in the interim ^18^F-fluoride PET/CT studies, 12 (35.3%) of whom were categorized as true-negative cases (Figure 3), the remaining patient (3.0%) being categorized as a false-negative case because the increased uptake noted on the interim ^18^F-fluoride PET/CT (when compared with that observed in the baseline study) was actually due to a flare phenomenon (Figure 4). Therefore, the responses were categorized as true positive in 11 cases (32.4%), false positive in 10 (29.4%), true negative in 12 (35.3%), and false negative in 1 (2.9%).

**Figure 5:**
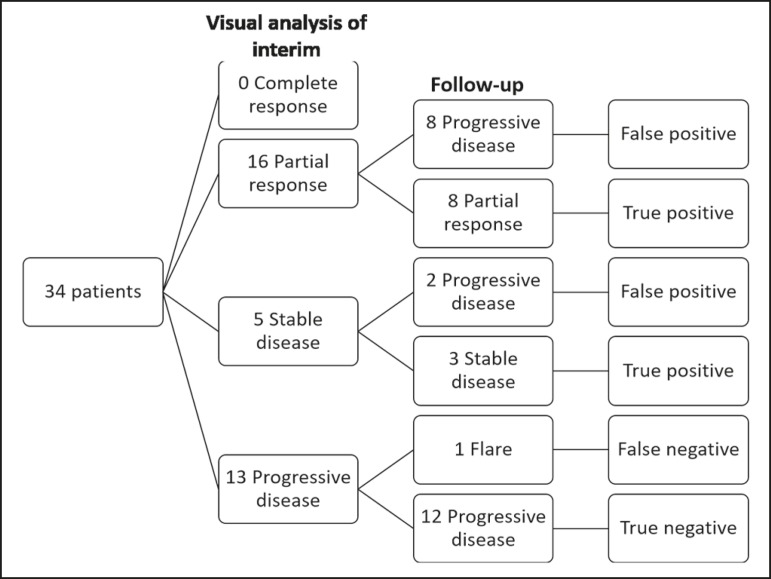
Visual analysis and evolution of the 34 patients.

The interim ^18^F-fluoride PET/CT study was found to have a sensitivity of 91.6%, a specificity of 54.5%, a positive predictive value of 52.4%, a negative predictive value of 92.3%, and an accuracy of 67.6% (Figure 5). For distinguishing between responders and nonresponders, a reduction in the ALP level had a sensitivity of 38% and a specificity of 85% when the follow-up parameters were taken as the reference.

### Quantitative analysis of interim ^18^F-fluoride PET/CT

**Figure 6:**
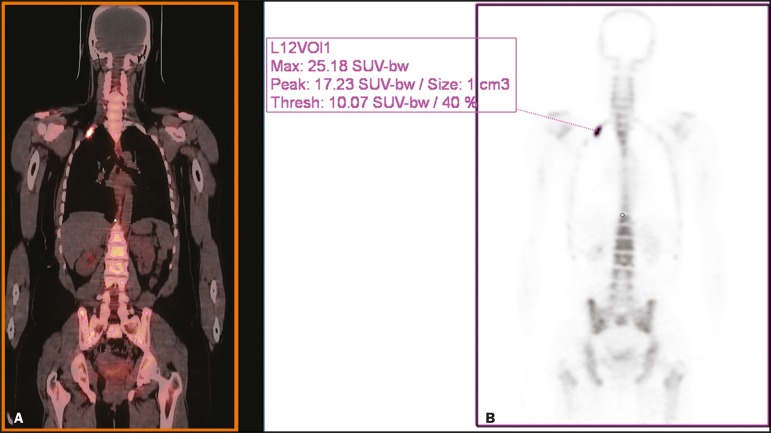
Example of determination of TLF_10_ and FTV_10_. **A:** A semi-automatic VOI (orange rectangle) is placed within the whole-body maximum intensity projection image. A threshold SUV_max_ of 10 is then established as the cut-off to separate normal bone from abnormal bone. Consequently, the software will automatically delineate only SUV_max_ regions above the set threshold of 10, defining the VOI with an isocontour threshold set at 41% of the SUV_max_. After all regions have been defined, a careful inspection should be performed to exclude all non-tumor-related VOIs. The sum of all the VOIs outlined with the SUV_max_ of 10 provides the FTV_10_. To obtain the TLF_10_, the FTV_10_ is multiplied by the SUVmean_10_ (VOI_10_ × mean_10_), which is also measured in milliliters. **B:** In this particular example, the patient had only one lesion with an SUV_max_ higher than 10, which corresponded to a rib metastasis with an SUV_max_ of 25. The TLF_10_ was 65.8, and the FTV_10_ was 4.2.

Figure 6 illustrates the quantitative method employed to obtain the TLF_10_ and FTV_10_ values. Spearman's correlation coefficient showed that the %TLF_10_ and %FTV_10_ values correlated strongly with each other (rho = 0.95). Therefore, only the %TLF_10_ values were applied to subsequent analyses. The median TLF_10_ was 7374 (range, 391-46,550) in the baseline ^18^F-fluoride PET/CT study and 5632 (range, 486-30,200) in the interim study.

At the end of the follow-up period, 32 (94%) of the patients had progressed and 17 (53%) had died (Table 2). The average time to progression was 4.7 ± 2.9 months (median, 3.1 months; range, 0.9-12.1 months), and the most common type of progression was metastasis to the bone (in 39.1%), followed by nodal metastases (in 25.0%) and visceral metastases (in 21.9%).

**Table 2:** %TLF_10_ variation and outcome measures.

		OS			PFS			TTBE			BMF	
Patient	%TLF_10_	Status	Months		Status	Months		Status	Months		Status	Months
1	46.9%	Alive	17.4		Yes	3.0		No	17.4		No	9.9
2	-44.0%	Alive	11.8		Yes	2.8		No	11.8		No	8.8
3	-22.4%	Alive	18.7		Yes	11.9		No	18.7		No	13.2
4	-70.0%	Alive	11.4		Yes	0.9		No	11.4		No	8.0
5	-7.3%	Deceased	8.6		Yes	6.5		Yes	6.5		No	6.6
6	-33.0%	Deceased	8.7		Yes	3.4		No	8.7		Yes	3.4
7	-35.1%	Alive	9.0		Yes	5.2		No	9.0		Yes	7.7
8	-33.8%	Deceased	10.4		Yes	3.0		No	10.4		No	6.7
9	17.8%	Deceased	11.1		Yes	2.8		No	11.1		No	10.5
10	-51.3%	Deceased	9.0		Yes	2.2		Yes	2.2		No	4.5
11	-9.8%	Alive	13.9		Yes	2.8		Yes	6.2		No	9.5
12	39.6%	Alive	3.7		Yes	2.8		No	3.7		No	3.7
13	-24.5%	Deceased	6.0		Yes	3.1		No	6.0		Yes	4.5
14	-17.1%	Alive	10.7		Yes	7.7		No	10.7		No	7.6
15	34.6%	Alive	11.5		Yes	8.1		No	11.5		No	10.3
16	-22.4%	Alive	15.4		Yes	9.3		No	15.4		No	11.4
17	14.2%	Alive	4.8		Yes	2.8		Yes	2.8		Yes	4.3
18	-20.4%	Alive	11.9		Yes	7.1		No	11.9		No	6.1
19	-25.6%	Alive	9.5		Yes	4.2		No	9.5		No	7.9
20	-8.8%	Alive	13.9		Yes	8.1		No	13.9		No	11.7
21	16.1%	Deceased	8.7		Yes	2.8		No	8.7		No	3.7
22	65.5%	Alive	16.3		No	12.1		No	16.3		No	8.1
23	-26.0%	Deceased	4.7		Yes	1.8		No	4.7		No	3.5
24	-66.3%	Alive	17.7		Yes	10.0		Yes	4.5		No	12.1
25	-11.6%	Deceased	5.9		Yes	2.0		No	5.9		Yes	5.1
26	-18.9%	Deceased	5.0		Yes	2.8		No	5.0		Yes	2.8
27	104.0%	Deceased	12.1		Yes	3.2		No	12.1		Yes	11.9
28	-43.8%	Deceased	8.2		Yes	5.1		Yes	8.2		No	5.1
29	-28.8%	Alive	9.6		Yes	4.6		No	9.6		No	4.6
30	19%	Deceased	8.2		Yes	5.0		No	8.2		No	5.0
31	65.5%	Deceased	6.8		Yes	2.8		No	6.8		Yes	6.4
32	-84.5%	Deceased	5.8		Yes	2.8		No	5.8		Yes	5.6
33	-62.8%	Alive	9.3		No	4.9		No	9.3		No	4.8
34	8.4%	Deceased	5.4		Yes	3.1		No	5.4		No	4.2

In our study sample, the %TLF_10_ on the interim ^18^F-fluoride PET/CT was not able to predict OS (*p* = 0.6320; HR = 0.753; 95% CI: 0.236-2.401) or PFS (*p* = 0.5908; HR = 1.248; 95% CI: 0.557-2.797). Six patients had a bone event, and %TLF_10_ was also unable to predict the TTBE (*p* = 0.5114; HR = 1.588; 95% CI: 0.399-6.312). Nine patients developed BMF after ^223^RaCl_2_, and %TLF_10_ was also not a significant univariate predictor of the odds of developing that condition (*p* = 0.6071; HR = 1.401; 95% CI: 0.387-5.070). We found that OS did not correlate with the SUV_max_ (*p* = 0.7989), any nodal disease (*p* = 0.1342), or visceral disease (*p* = 0.1496).

## DISCUSSION

We have demonstrated that an interim ^18^F-fluoride PET/CT study is unable to predict outcomes after ^223^RaCl_2_ therapy. Novel therapies for osteoblastic metastases, including ^223^RaCl_2_ therapy, are costly, and it is therefore important to establish a diagnostic test to predict responses to these new, expensive treatments. In one study evaluating treatment responses after six cycles of ^223^RaCl_2_ in ten patients^(^13^)^, conventional bone scintigraphy demonstrated that increased areas of uptake were due not only to treatment response but also to reparative bone changes after therapy (a flare response).

Previous studies have shown that a baseline ^18^F-fluoride PET/CT study plays a prognostic role in patients with breast or prostate cancer treated with ^223^RaCl_2_^(^1^,^^14^^)^. However, ^18^F-fluoride PET/CT is not traditionally used in evaluating the response to any therapy, because the process of bone healing involves an osteoblastic reaction than can increase ^18^F-fluoride uptake, as in conventional bone scintigraphy^(^15^)^. Because of comparable pharmacokinetics between ^223^RaCl_2_^(^2^)^ and ^18^F-fluoride^(^16^)^, we hypothesized that ^18^F-fluoride would be able to evaluate osteoblastic metastases before, during, and after ^223^RaCl_2_ therapy.

In our study sample, the interim study demonstrated that a decrease in uptake was generally due to a response (partial or stable disease) to therapy. However, we find it interesting that, in six (17.6%) of the patients, the decreased uptake was caused by extensive tumor infiltration of the bone marrow, ultimately leading to BMF. To our knowledge, there have been no previous studies describing the latter imaging pattern (caused by BMF) in interim studies. In contrast, although the interim study was able to demonstrate that increased uptake was due to progression, that pattern of uptake was in fact a flare phenomenon in one case. This increased uptake most likely occurred because of the bone healing process after successful ^223^RaCl_2_ treatment, which involves an osteoblastic reaction. In the subset of patients in which the flare phenomenon occurred, the CT portion of the study revealed reparative changes with increased extent of the sclerotic lesions. However, even on CT, it was not possible to determine which patients were progressing and which were responding. Although we hypothesized that bone levels of ALP could help evaluate patient outcomes, it demonstrated higher specificity and lower sensitivity than did the interim ^18^F-fluoride PET/CT study.

Quantitative analyses of ^18^F-fluoride PET/CT images have been conducted to assess its role in predicting outcomes, by determining the peak SUV_max_ values of bone metastases. Apolo et al.^(^17^)^ performed ^18^F-fluoride PET/CT after 6 and 12 months of standard therapy in prostate cancer patients, reporting that progression was associated with SUV increases of more than 57%, as well as that a greater increase in SUV was associated with worse survival. Yu et al.^(^18^)^ evaluated responses to therapy with dasatinib using SUV_max_ in five target lesions on ^18^F-fluoride PET/CT and detected only a borderline correlation with PFS; the changes also correlated with the ALP level. Another study, involving only five patients, showed a reduction in SUV_max_ at 6 and 12 weeks after the use of ^223^RaCl_2_^(^19^)^. In our population, the SUV_max_ did not correlate with OS. Although the above mentioned studies performed ^18^F-fluoride PET/CT for therapeutic evaluation, its precise role in determining the early response to therapy has yet to be extensively studied, especially in terms of assessing survival as an end point.

The reported frequency of the flare phenomenon in prostate cancer patients undergoing conventional bone scintigraphy ranges from 6% to 25%^(^20^,^^21^^)^. Although the flare phenomenon has also been described in patients undergoing ^18^F-fluoride PET/CT^(^15^)^, there have been no reports of its frequency in patients treated with ^223^RaCl_2_ and undergoing ^18^F-fluoride PET/CT. Although we identified the flare phenomenon on ^18^F-fluoride PET/CT in only a small proportion of our patient sample (3%), that proportion is probably higher than in conventional bone scintigraphy, given the greater sensitivity of PET/CT. The most likely explanation for the fact that the frequency of the flare phenomenon was not higher is that our study sample was composed of patients with extensive disease, in whom the likelihood of progression is greater than is that of a response to therapy. In addition, the number of patients might have been insufficient to detect this phenomenon.

In our patient sample, the interim ^18^F-fluoride PET/CT (%TLF_10_) after three cycles of ^223^RaCl_2_ was not able to predict OS, PFS, TTBE, or BMF. These findings are quite similar to those of a previous study, involving ten prostate cancer patients treated with ^223^RaCl_2_^(^22^)^, although the interim ^18^F-fluoride PET/CT studies were performed at different time points: at baseline; after one (or two) cycles of ^223^RaCl_2_; and at the end of treatment. A correlation with outcome was only noted between baseline and end-of-treatment ^18^F-fluoride PET/CT results were found to correlate with outcomes, as previously reported^(^1^)^.

One major limitation of our study was the relatively small number of patients. We believe that the interim ^18^F-fluoride PET/CT could have potential for the prediction of BMF, given that 6 of the 9 patients who evolved to BMF showed a reduction in uptake. However, due to the small sample size, those results were not significant. Another limitation was the fact that it was not possible to obtain histological confirmation in the patients who showed progression. Although the ^18^F-fluoride PET/CT images were acquired in different scanners, the same software was employed in all quantifications, guaranteeing uniformity in the metrics.

To our knowledge, this is the first study to evaluate the role of interim ^18^F-fluoride PET/CT in predicting the response to ^223^RaCl_2_ therapy, using quantitative methods to determine the skeletal tumor burden. It would be interesting to know whether these findings could be replicated in other populations, such as that of breast cancer patients treated with ^223^RaCl_2_.

## CONCLUSION

In prostate cancer patients undergoing ^223^RaCl_2_ therapy, interim ^18^F-fluoride PET/CT performed after three cycles of ^223^RaCl_2_ does not seem able to predict outcomes. It also appears to be unable to differentiate a flare response from progressive disease, and we therefore discourage the use of interim ^18^F-fluoride PET/CT to evaluate the response to ^223^RaCl_2_ therapy in prostate cancer patients. There is a need for studies involving a larger number of patients and patients with other types of cancer, in order to verify our findings.
